# Targeted sequencing reveals the somatic mutation landscape in a Swedish breast cancer cohort

**DOI:** 10.1038/s41598-020-74580-1

**Published:** 2020-11-09

**Authors:** Argyri Mathioudaki, Viktor Ljungström, Malin Melin, Maja Louise Arendt, Jessika Nordin, Åsa Karlsson, Eva Murén, Pushpa Saksena, Jennifer R. S. Meadows, Voichita D. Marinescu, Tobias Sjöblom, Kerstin Lindblad-Toh

**Affiliations:** 1 Science for Life Laboratory, Department of Medical Sciences, Uppsala University, Uppsala, Sweden; 2 Science for Life Laboratory, Department of Immunology, Genetics and Pathology, Rudbeck Laboratory, Uppsala, Sweden; 3 Science for Life Laboratory, Department of Immunology, Genetics and Pathology, Rudbeck Laboratory, Uppsala, Sweden; 4 Department of Veterinary Clinical Sciences, Faculty of Health and Medical Sciences, University of Copenhagen, Copenhagen, Denmark; 5 Science for Life Laboratory, Department of Medical Biochemistry and Microbiology, Uppsala University, Uppsala, Sweden; 6 Department of Clinical Pathology and Genetics, Sahlgrenska University Hospital, Gothenburg, Sweden; 7 Broad Institute of MIT and Harvard, Cambridge, MA USA

**Keywords:** Cancer genomics, Breast cancer

## Abstract

Breast cancer (BC) is a genetically heterogeneous disease with high prevalence in Northern Europe. However, there has been no detailed investigation into the Scandinavian somatic landscape. Here, in a homogeneous Swedish cohort, we describe the somatic events underlying BC, leveraging a targeted next-generation sequencing approach. We designed a 20.5 Mb array targeting coding and regulatory regions of genes with a known role in BC (*n* = 765). The selected genes were either from human BC studies (*n* = 294) or from within canine mammary tumor associated regions (*n* = 471). A set of predominantly estrogen receptor positive tumors (ER +  85%) and their normal tissue counterparts *(n* *=* 61) were sequenced to ~ 140 × and 85 × mean target coverage, respectively. MuTect2 and VarScan2 were employed to detect single nucleotide variants (SNVs) and copy number aberrations (CNAs), while MutSigCV (SNVs) and GISTIC (CNAs) algorithms estimated the significance of recurrent somatic events. The significantly mutated genes (*q ≤ *0.01) were *PIK3CA* (*28*% of patients), *TP53* (*21%*) and *CDH1* (*11%*). However, histone modifying genes contained the largest number of variants *(KMT2C* and *ARID1A*, together *28%)*. Mutations in *KMT2C* were mutually exclusive with *PI3KCA* mutations (*p ≤ 0. 001*) and half of these affect the formation of a functional PHD domain. The tumor suppressor *CDK10* was deleted in 80% of the cohort while the oncogene *MDM4* was amplified. Mutational signature analyses pointed towards APOBEC deaminase activity (*COSMIC signature 2*) and DNA mismatch repair (*COSMIC signature 6*). We noticed two significantly distinct patterns related to patient age; *TP53* being more mutated in the younger group (29% vs 9% of patients) and *CDH23* mutations were absent from the older group. The increased somatic mutation prevalence in the histone modifying genes *KMT2C* and *ARID1A* distinguishes the Swedish cohort from previous studies. *KMT2C* regulates enhancer activation and assists tumor proliferation in a hormone-rich environment, possibly pointing to a role in ER + BC, especially in older cases. Finally, age of onset appears to affect the mutational landscape suggesting that a larger age-diverse population incorporating more molecular subtypes should be studied to elucidate the underlying mechanisms.

Breast cancer (BC), the most commonly diagnosed malignancy and a leading cause of death in women worldwide, has among the highest reported incidence rates in Northern Europe^1^. Clinically, BC is classified based on the initial morphological assessment of the tumor (type, size, grade, lymph node status), and more recently, on the basis of the expression of receptors of estrogen (ER), progesterone (PR) and human epidermal growth factor 2 (ERBB2/HER2). Receptor expression is part of a routine tumor assessment and provides a rough separation of BC into three subtypes (ER + , HER2 + and triple negatives), serving as a prognostic marker and assisting in the selection of treatment^2^. Overall, 75% of BC tumors appear to be ER + but this observation alone is not sufficient to accurately predict the response to treatment and the disease progression^3^.

BC is a genetically complex and heterogeneous disease where explained heritability (family history, *BRCA1/2* mutations) accounts for only a small percentage (~ 20%) of the familial cases in the general population^4,5^, and cannot elucidate BC geographical distribution, molecular subtypes, and variability in treatment response. Studies on extensive cohorts, facilitated by the tools and methods of the genomics era, have revealed the genetic heterogeneity in breast cancers. Hereditary cases are in part explained by germline mutations in *BRCA1* and *BRCA2* ^6^, and many markers have been identified that confer risk to BC^7,8^. Cancer genetic research has recently been focusing on the characterization of the driver somatic mutations and attempting to link driver mutations to clinical phenotypes, providing insights on therapeutic regime selection and treatment response. As a result of these efforts, numerous somatic events, including CNAs and high-frequency substitution and insertion/deletion (indels), have been revealed. Some of the most frequent somatic events include mutations (*TP53, PIK3CA, CDH1, AKT1, GATA3, MAP2K7*, *MYC)*, duplications (*ERBB2)* and deletions (*PTEN* or *MAP2K4)*. Mutation rates are correlated with BC tumor subtype, and vary across study designs and sample sizes^9–12^.

To date, cancer somatic landscapes have not been compared across countries or specifically described in different ethnic populations within Europe. Here, we present an effort to characterize the somatic landscape of a Scandinavian BC population, utilizing a targeted array designed based on both human and dog genetic studies. Specifically, we sequenced a Swedish BC cohort of homogeneous ancestry to identify potential differences to other ethnical groups and BC molecular subtypes. We report the significantly mutated genes (SMGs), recurrent CNAs and the mutational signatures and we highlight age-dependent differences in the tumors.

## Methods

### Patient sample collection and processing

This study utilized material from 65 BC patients undergoing surgery at Uppsala University Hospital after obtaining signed informed consent from all patients in accordance with ethical guidelines (Additional File 1: S. Table 1). The selection of cases was based on four criteria: i) tumor type being either lobular (LC) or ductal carcinoma (DC), ii) no neoadjuvant treatment, to avoid drug-induced mutations, iii) sufficient tumor DNA (> 0.9 µg), and iv) sufficient normal tissue DNA (histopathologically available, > 0.1 µg). High molecular weight DNA was extracted from both the primary tumor and the adjacent normal breast tissue and was hybridized to the liquid custom array using an in-house optimized protocol^13^. A Gaussian finite mixture model fitted by EM algorithm was applied to the data through the R package *mclust* in R v. 3.5.0^14^, where all R processes of the study were performed, to identify significant age of onset clustering within our cohort^15^.

**Table 1:** Descriptive statistics of Swedish patients and tumor sets.

Characteristics	Patient % (count)
**PATIENTS**	
**Age**	
**Mean age (years)**	
67.4	
**Range (years)**	
43–96	
**Group**	
< 70 years	62% (38)
> 70 years	38% (23)
**Ancestry**	
European	100% (61)
**Site**	
Primary	100%(61)
**Treatment**	
No neoadjuvant	100%/61)
**TUMORS**	
**Hormone-receptor**	
ER +	85% (52)
PR +	77% (47)
HER2 +	11%(7)
ER + /PR +	66% (40)
ER-/PR-/HER2-	3% (2)
**Histopathology**	
Lobular	7% (4)
Ductal	92% (56)
Ductal-in situ	13% (8)
**Tumor fraction**	
< 50%	18% (11)
51–60%	30% (18)
61–70%	22% (14)
71–85%	30% (18)

*ER* estrogen receptor, *PR* progesterone receptor, *HER2* human epidermal growth factor 2.

### Array design and NGS

The design of the custom 20.5 Mb SeqCap EZ Choice XL enrichment array (Roche NimbleGen) focused on a specific set of targets encompassing 765 genes (Additional File 1: S.Table 2). The selection of genes was based on two lines of evidence i) genes with known or suspected role in human BC or in cancer pathways (*n* = 294) and ii) human genomic regions analogous to loci associated with canine mammary tumor (CMT) in a genome-wide association studies (GWAS) in Swedish non-spayed English Springer Spaniels^16^. The initial hg18 array design included the coding regions of these genes, 5′ UTRs, 3′UTRs, their potential promoter regions (within 2 kb of the transcription start sites) and splice sites (20 bp of intronic sequences adjacent to exons). Furthermore, regions of evolutionary conservation (SiPhylod score > 7 ^17^) within 100 kb up- or downstream of those targets were also included. On the pathway level, the gene targets of the array represented genes in nearly all known pathways in cancer. On the gene level, the array captured genes (some partially) with an established role in cancer (*n* = 150) but also genes defined as putative cancer drivers (*n* = 167) by the Network of Cancer Genes v5.0^18^. Pools were generated, each containing four barcoded individual libraries, and those with a concentration of less than 115 ng/µl (*n* = 60 normal samples) were amplified for four cycles (Thermo Fisher Scientific primers), before hybridization with the liquid array. Successful hybridization was assessed with a qPCR target-enrichment control, which was followed by 100 bp paired-end read sequencing with Illumina HiSeq 2500 (v3). Each sequencing lane consisted of pools where tumor material amount was double than the normal tissue amount.

### Somatic variant calling and filtering pipeline

The pipeline for the analysis of the data is summarized in Additional File 2: SF1. The target coordinates were converted from hg18 to hg19 using the LiftOver tool (accessible in the UCSC browser^19^), and the raw reads for each sample were mapped to hg19 using the *mem* function of the BWA package 0.7.12^20^. In the preprocessing steps, we applied GATK v3.5.0 best practices including marking duplicate reads (Picard 1.92^21^), local read realignment and base quality score recalibration (BQSR^22–24^). Before performing the somatic variant calling, we calculated the amount of normal contamination in the tumor within a tumor/normal pair using ContEst^25^, and estimated the percentage of oxidative stress artifacts in our data (Picard 1.92^21^). Also, off target reads from all normal sequences were used to identify potential ancestry outliers (*n* = 1) using LASER^26^ (Additional File 3: SF2). In addition, to identify potential substructure within the cohort we performed identity by state (IBS) clustering on the basis of autosomal data derived from the normal tissues. First, we removed the high linkage disequilibrium (LD) regions identified in the European population from our initial set of mutations^27^. Then the visualization of the clusters identified three outlier samples that were removed from further analysis (Additional File 3: SF3). After these steps, normal samples that passed the quality and ancestry control were grouped to form a panel of normals (PON) that represented the germline variation in the tumors when calling the somatic variants. All in all, after Illumina HiSeq 2500 v3, ancestry and quality control, 61 tumor-normal pairs (Additional File 1: S.Table 1) were retained in the study.

Somatic point mutations were called with MuTect2^28^ from GATK v3.6.0, while indel discovery was performed using the heuristic algorithm of VarScan2 v2.4.2^29^. Removal of oxidative artifacts was performed with a custom-made Python script^30^. The somatic variants were further filtered to remove i) germline contamination by subtracting variants found in PON (*n* > 2), ii) variants where the alternate allele was present or recorded as multievent in the normal counterpart, iii) germline risk variants from the dbSNP white-listed as per MuTect2 instructions, iv) homologous mapping events v) tri-allelic sites, vi) low log-odds of an event in the tumor (*t_lod*) and vii) short tandem repeat contractions. The remaining somatic variants were inspected with the Integrative Genome Viewer (IGV) and their functional annotation was performed with Oncotator^31^.

### Identification of recurrently and significantly mutated genes

Vcf files containing annotated somatic variants were converted to mutation annotation format (maf) files (Additional File 1: S.Table 3) and used as input for the *maftools* package^32^ in R, to summarize, analyze and visualize the mutation set. The recurrently mutated genes were identified using *maftools* based on the number of samples carrying mutations in their coding sequence (including UTRs). SMGs were retrieved by using the maf file as input for MutSigCV v.1.3.01 algorithm^33^, within the MATLAB (R2018α) environment^34^ based on estimating genes that were mutated more often than expected, considering background mutation rate (BMR) as implemented in the algorithm. To account for false discovery rate (FDR), a significance threshold (*q* < 0.01) was selected and genes exceeding this value were assigned as SMGs.We calculated the mutation exclusivity within the top three mutated genes with binomial tests in R (i.e. *t-test* and x^2^ tests for independence). To investigate the physical position of the mutations and their potential impact on the protein structure, we plotted them visually on the longest RefSeq transcript using *maftools,* in R. Variant allele fraction (VAF) was calculated as the percentage of reads observed at a specific variant divided by the overall coverage at the variant position and plotted to identify the clonal status of the top mutated genes. Putative driver genes were identified as the genes containing mutations with high VAF (VAF > 15%) in their coding sequence.

### CNAs detection and recurrent copy events identification

We used VarScan2 to identify somatic CNAs. By directly comparing the normalized sequence depth at positions with at least 20 × coverage, the tool returned the raw copy number calls. These were subjected to circular binary segmentation (CBS), to delineate segments by copy number and to categorize major regions with alterations. To merge similar adjacent events, a Perl script was used. Significantly amplified or deleted genomic regions were identified using the GISTIC software (Genomic Identification of Significant Targets in Cancer^35^). All aberrations were annotated with a G-score (amplitude of the event) and the frequency of their occurrence. For each significant region that passed the significance threshold (q-value < 0.02), we identified a “peak region” and a “wide peak” and also estimated whether the aberration was a focal or broad event. S. Table 4 lists the top recurrent aberrations found in our cohort and the genes present within their genomic coordinates. Visualization of the GISTIC results and CNAs enrichment analysis between phenotype groups was performed in R.

### Mutational signatures and mutated pathways

Taking into account all the possible mutated trinucleotides in every position, we used the *BS genome* package^36^ to build a 96-trinucleotide matrix for each SNV^37,38^ and then extracted the most probable mutational signatures. Using non-negative matrix factorization (*NMF package* in R), we contrasted the identified signatures against known and validated signatures^37,39^ and identified the best match as the one with the highest cosine similarity value. Additional File 4: SF4 shows the NMF results visualized in R. Furthermore, we identified which pathways as defined in TCGA^40^ data are mutated in our cohort and computed the fraction of affected genes per pathway.

### Ethics approval and consent to participate

All work was performed in accordance with ethical guidelines and the study was approved by the Ethical Review Board in Uppsala (2007/116).

## Results

We collected a total of 65 tumor-normal pairs from well-characterized patients with BC and performed targeted sequencing using a 20.5 Mb custom array. After quality control (QC) that included ancestry analysis and IBS clustering (SF2 & 3), a total of 61 cases of which 57 (92%) were DC and 4 (6%) were LC (Table 1), were retained for further analysis. In the retained set, 85% were ER-positive, 77% were PR-positive while 66% were both ER- and PR-positive. 63% were HER2 negative (Table 1) and two samples were identified as triple negative. When examining the distribution of the age of onset for our cohort, we observed that it had a bimodal aspect. In consensus with this observation, a Gaussian finite mixture model fitted by the EM algorithm for mixture estimation showed that our cohort could be divided in two clusters based on age. Therefore, we divided the cohort into two separate age groups i) under 70 years old (n = 38, mean 57.5 yr) and ii) above 70 years old (n = 23, mean 83.1 yr) (Additional File 5: SF5).

The mean target coverage was 140× (range 50–290×) for the tumor samples and 85× (range 28–147×) in the normal samples. The data from the normal tissue was used to check the ancestry of the samples to ensure that the cohort was of a homogeneous European ancestry (Additional File 3: SF2 B) and also underwent IBS clustering to ensure that there was no cryptic substructure (Additional File 3: SF3). After ancestry and quality control, somatic events were called and used for downstream analysis. The somatic SNV set consisted of 3635 somatic point mutations (SPMs) and 2534 indels while the CNAs set included 225 amplifications and 3238 deletions.

**Figure 1:**
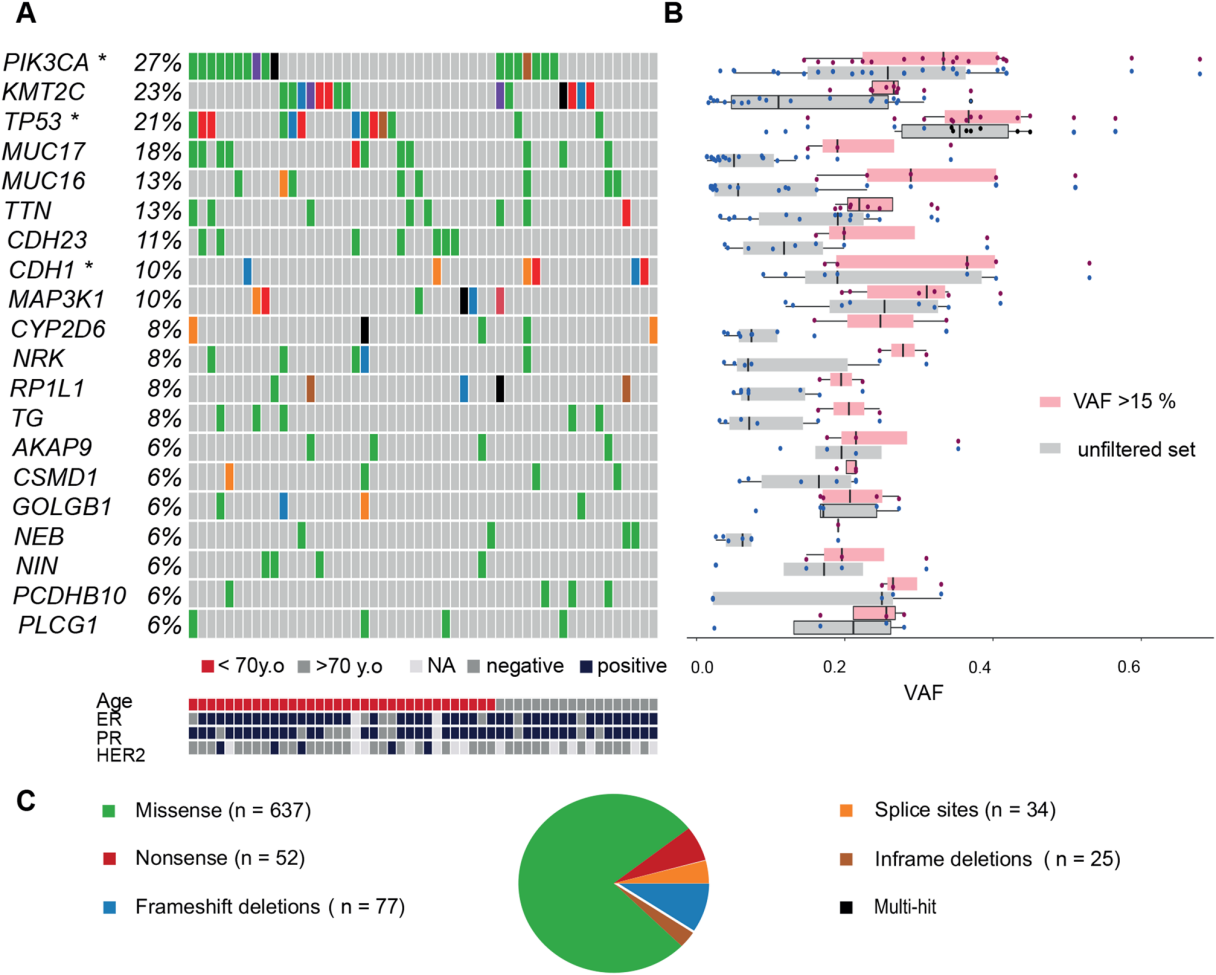
Onco-plot of somatic SNVs in the Swedish breast cancer cohort. (**A**) Brick plot of somatic mutations in frequently and significantly mutated genes (SMGs). Each horizontal lane describes a single gene and the vertical lines represent different samples while different colors indicate the type of mutations (described in 1C). Age and hormone receptor status in the Swedish BC cohort are schematically described with patients ordered by age (under 70 years old shown in red, or above 70 years old shown in gray) and the receptor expression (ER, PR, HER2) is also shown (blue is positive expression, grey is negative). SMGs (*PIK3CA*, *TP53* and *CDH1*) are marked with *. *KMT2C* contains mutations in 23% of the cohort. *PIK3CA* and *KMT2C*-mutated individuals are evenly distributed between the two age groups and 85% of *TP53*-mutated individuals were found in the younger group. In addition, *CDH23* mutations were found exclusively in the younger age group. (**B**) Boxplot of the mutation variant allele fraction (VAF) per gene before (grey) and after VAF filtration 15% (light pink). The amount and clustering of variants is shown with red dots depicting the filtered set and the black dots the unfiltered set. The SMGs contain clusters of mutations with high VAF. (**C**) Breakdown of the different type of somatic SNVs and InDels. Missense mutations were most commonly observed (green, n = 637), followed by frameshift InDels (blue), and nonsense mutations (red). Notably, *CDH1* contained more splice site mutations (orange).

The samples exhibited a relatively equal number of coding variants (median 9, mean 13, SD 20). We found an overall mutation rate of 2.7 mutations per Mb and a mean mutation rate of 0.50 mutations per Mb in coding regions. This was similar to the rate previously observed (0.61 in TCGA^41^), (Additional File 5: SF6). The vast majority (87%) of the 826 coding mutations (637 missense, 52 nonsense, 1 nonstop, 77 frameshift, 25 in-frame indels and 34 in splice sites) (Fig. 1C) had been previously observed in BC and other cancers.

### SMGs, driver mutations and mutation co-occurrence in the Swedish cohort

To reveal which genes were frequently mutated in the Swedish BC cohort, we examined the set of coding somatic SNVs and indels per gene, calculating their mutation rates. The most frequently mutated genes were *PIK3CA* (28%), *KMT2C* (23%) *and TP53* (21%) (Fig. 1A) followed by three genes coding for large proteins (*MUC16*, *MUC17*, *TTN* at 13–21%) cadherin genes (*CDH23, CDH1*, ~ 11%) and *MAP3K1* (10%). Next, we used MutSigCV to determine which genes were mutated above the BMR which identified *PIK3CA, TP53* and* CDH1* (marked with * in Fig. 1A, q-value < 0.01). Upon closer investigation of the SMGs, *PIK3CA* and *TP53* contained many missense mutations while *CDH1* had more splice site mutations. Notably, *KMT2C* (*MLL3*) while mutated in 23% of the samples was not identified as an SMG, but it contained many frameshift and splice site mutations, suggesting involvement in BC.

In order to identify putative driver mutations, mutations with a low VAF needed to be removed. Thus, we performed VAF filtering (VAF > 15% were kept, Fig. 1B), based on the hypothesis that the driver mutations exhibit a higher VAF. After filtering and following the same methodology, the retained coding mutations (n = 456) revealed a different ranking of the genes based on their mutation rate (Additional File 6: SF7A); *MUC16* and *MUC17* were down-ranked whereas *KMT2C* was up-ranked; *TP53* contained more mutations than *KMT2C* above the VAF cutoff and was the second most frequently mutated gene. Looking closely at the VAF clusters within the genes, we report that *CDH1*, *TP53* and *PIK3CA* had the highest VAF (36–39%) followed by *MAP3K1*, and *KMT2C* (Additional File 6: SF7B). The MutSigCV algorithm was utilized in this filtered somatic SNVs set and still retrieved *PIK3CA, TP53, CDH1* as SMGs (*q*-value < 0.01) and possible drivers (marked with * in Additional File 6: SF7A).

We next investigated if the array content stemming from canine association studies provided new information for the human study by examining the mutations in it. Overall, CMT genes contained coding mutations in 4% of the samples and the main CMT region contained among others *CDH23* on chr10, mutated in 11% of the patients. The overlap of genes from the earlier CMT study with the genes from human studies, increased over time with the development and analysis of many somatic exomes and whole genomes^42^.

We observed that there were no samples containing mutations in all of the top three genes *PIK3CA*, *KMT2C* and *TP53* at the same time, prompting us to examine whether there was non-independence between mutations in these genes. We noted that in the *PIK3CA*-mutated samples there is an observed co-occurrence with *KMT2C* mutations in two samples. However, that still represents a significant underrepresentation of the double-mutants, compared to the expectation if mutations are found independently (*x*^2^* test p value* = 1.3 × 10^–3^). In addition, there was no consistent proof that *PIK3CA* or *KMT2C* was mutated earlier than the other, as the VAF of the mutations was almost the same in the small number of samples with overlapping mutations. A similar observation was made for the *TP53* mutations in the *PIK3CA*-mutated samples where four samples contained mutations in both genes (*x*^2^* test p-value* = 6.9 × 10^–5^). We hypothesize that the mutations emerged in the same cancer progression stage since they shared the same VAF values. *KMT2C* mutations also showed a significant exclusivity with *TP53* mutations (*x*^2^* test p-value* = *4.1* × 10^–5^), co-occurring in three samples, while the lower *KMT2C* VAFs suggested that *KMT2C* was mutated after *TP53.*

### Recurrent deletion of *CDK10* and recurrent amplification of *MDM4*

**Figure 2:**
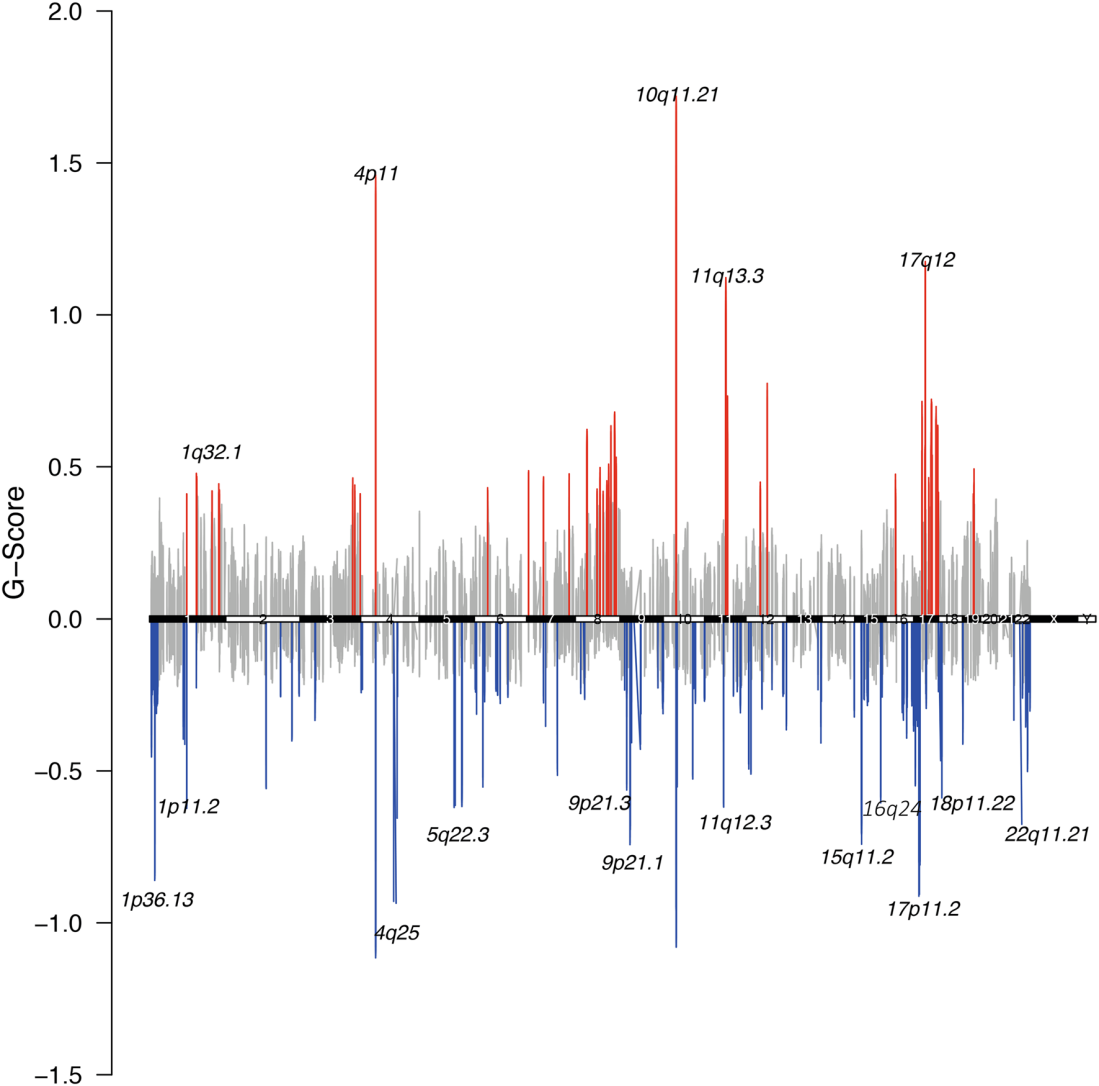
Recurrent copy number aberrations in the Swedish breast cancer cohort. Regions of recurrent copy number amplifications (red) and deletions (blue) in the targeted array were identified with GISTIC 3.0 (*q*-value < 0.2). Cytobands, across all the chromosomes, containing the top 20 recurrently altered regions are annotated and marked with pink vertical lines.

To identify CNAs, we used VarScan2 and circular binary segmentation. We identified 85,586 CNAs (mean 1,380 per sample) and found that deletions were more frequent than amplifications (*n*_*DEL*_ = *48,783; n*_*AMP*_ = *36,803*). We used GISTIC to investigate recurrent CNAs and found 21 significantly amplified and 46 significantly deleted regions (n = 67, 99% confidence, S.Table 5). Visualization of the copy number aberrations with highest G-score (Fig. 2) showed amplifications on chromosomes 4, 10 and 11 and deletions on chromosomes 4, 10 and 17. The focal CNAs contained several cancer relevant genes, such as the known breast cancer genes *EGF*, *CDKN2A* and *BAG1* and some were less established including *ZAR1* and *BMS1* (S. Table 5). The most recurrent deletion (16q24.3, found in 80% of cases), harbored the known BC tumor suppressor *CDK10*. At the same time, the most prevalent amplification (1q32.1) affecting 80% of cases), encompassed more than 100 genes including the BC oncogene *MDM4*^43^. Apart from chromosomes 1 and 16, amplifications or deletions were also observed on other chromosomes but these events were not observed in a large proportion of the samples (36–44%). Notably, the genes that contained the most SNVs (*PIK3CA*, *KMT2C*, *TP53* and *CDH1*) were not subject to copy number changes. However, when we examined all somatic events together (SNVs, indels, and CNAs), two genes on chromosome 6 were more frequently affected: *ZNF318* (Zinc Finger protein 318) and *ABCC10* (ATP binding Subfamily C member 10), (Additional File 6: SF8). From previous studies, ZNF318 is the best non-histone substrate to *HDAC8*^44^ that also preferentially deacetylates ARI1D1A, and interacts with ERRa in vitro^45^. In addition, ZNF318 was previously implicated in breast cancer with two positions: G1274R and N632S^46^. ABBC10, a transmembrane transporter protein is mostly expressed in ER + /HER2 + cancers^47^ and is implicated to docetaxel response^48^ while its downregulation was found to facilitate therapy response to paclitaxel, in ovarian cancer^49^. We also examined the status of known oncogenes and tumor suppressors for BC in our cohort and we observed that the tumor suppressors *CDH1, PTEN, BRCA2 and CDKN2A* had SNVs, with *CDH1* being the most mutated, showing splice and nonsense mutations and frameshift mutations in six samples. When considering the oncogenes, *PIK3CA* was not found to have copy number changes, but *PIK3C2B,* also a member of the phosphoinositide 3-kinase (PIK3) family and was fully targeted by the array, was amplified in 81% of cases. Interestingly, *PIK3C2B* did not contain coding mutations as *PIK3CA* did, but harbored only intronic mutations. The amount and different type of mutations show the importance of the PI3K pathway and the multiple ways its disruption affects BC initiation and progression. Also, we observed amplifications of *MYC* in 65%, *CCND1* in 52% and *ERBB2* in 47% of the samples. Notably *EGFR* and *KIT* showed a single mutation in one case each (data not shown).

### Location of mutations in the top genes within the Swedish cohort

**Figure 3:**
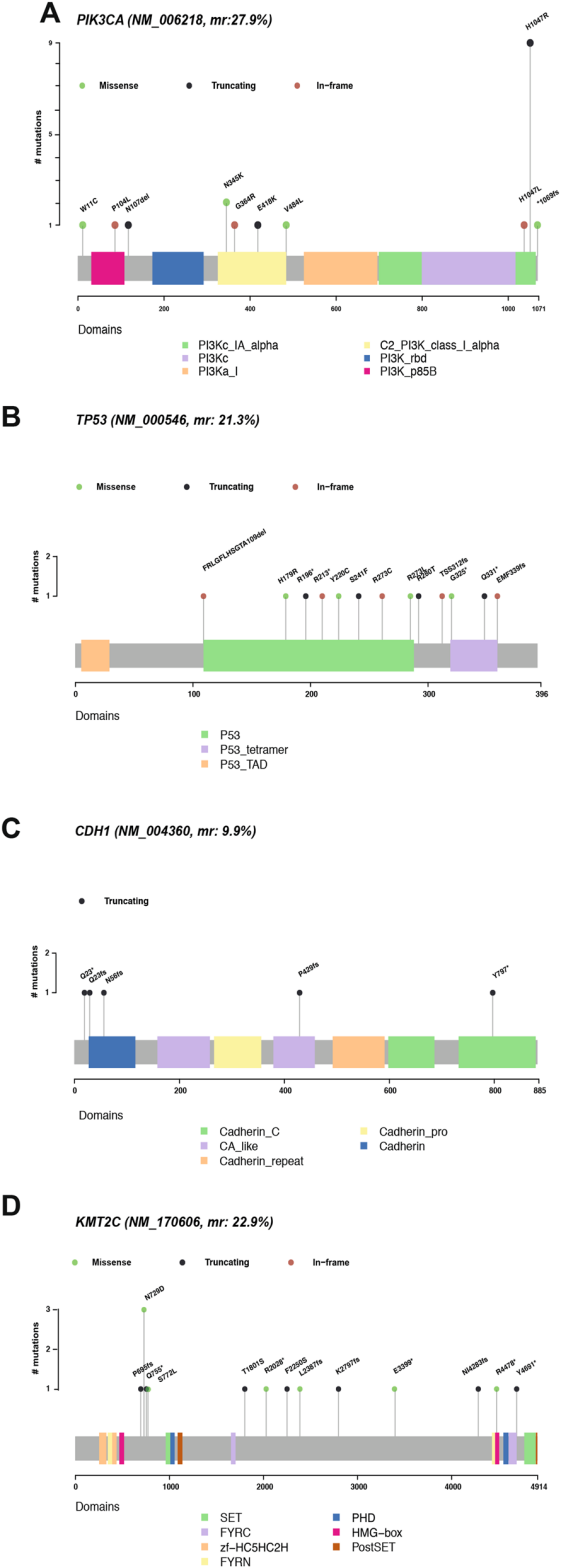
Somatic nucleotide level mutations in PIK3CA, TP53, KMT2C and CDH1. Illustrations of the somatic mutation distribution at the protein level. Gene level information was lifted over based on the longest transcript for each of the frequently mutated genes to capture the longest space. The x-axis shows the amino acid position and the y-axis the number of mutations observed in our cohort. Transcript and protein names are included (**A**) In PIK3CA (NM_006218, mr 27.8%, NP_006209.2), H1047R, a well-known BC hotspot, is replicated in the Swedish cohort. (**B**) In TP53 (NM_000546, mr 21.3%, NP_000537.3), no hotspots were identified in the cohort but a large proportion of the mutations were in the P53 domain. (**C**) Lollipop plot of CDH1 (NM_004360, mr 9.9%, NP_004351.1) in which three mutations were found around the cadherin domain. (**D**) In KMT2C (NM_170606, mr 22.9%, NP_733751.2), the PHD domain is not affected but mutations upstream of the domain may lead to a truncated protein without this domain.

To examine the potential role of the somatic SNVs in *PIK3CA, TP53, KMT2C and CDH1*, we examined their position in the encoded protein (Fig. 3). For PIK3CA, mutational hotspots have been reported in the helical (exon 9, E542K and E545K) and in the catalytic domain (exon 20, H1047R^50^).These variants lead the activation of the PI3K/AKT/mTOR pathway that promotes malignant formation and proliferation^51^. Here, we observed only the catalytic domain hotspot H1047R (Fig. 3A) that is characteristic of BC and associated with ER and node status^52^. *TP53* is a tumor suppressor gene that shows fewer somatic mutations in BC compared to other cancer types and with the majority of the BC mutations (75%) being germline missense substitutions^42^. Also, functional TP53 mutations usually occur in the P53 domain and less in the P53 tetramer part of the protein. In our cohort, we report mutations with high VAF distributed across that domain (Fig. 3B). From the known BC hotspots, we replicated a mutated amino acid position C220 and mutations on codons R273 and R280 that exhibit oncogenic properties^53^. *KMT2C* (*MLL3;* Fig. 3D) is a lysine methyltransferase part of the COMPASS family mainly creating H3K4me1 on enhancers^54,55^.The gene is located in a region on chromosome 7 that is often deleted in myeloid leukemia^56^ and proposed to act as a tumor suppressor. Correct recruitment of the KMT2C protein to its target genes is based on a functional extended PHD domain, where we did not find mutations. However, several observed upstream mutations (*n* = 4) would truncate KMT2C prior to the PHD domain. Also, we observed a missense hotspot at N729D (*n* = 1), a domain with unknown function. *CDH1,* a classical type I cadherin, has been identified as a BC tumor suppressor and when downregulated it facilitates a more aggressive cancer progression^57^. All five observed mutations were truncating; two splice variants and the remaining within cadherin, CA-like and cadherin-C domains (Fig. 3C).

### Mutational signatures and mutated pathways in Swedish BC

**Figure 4:**
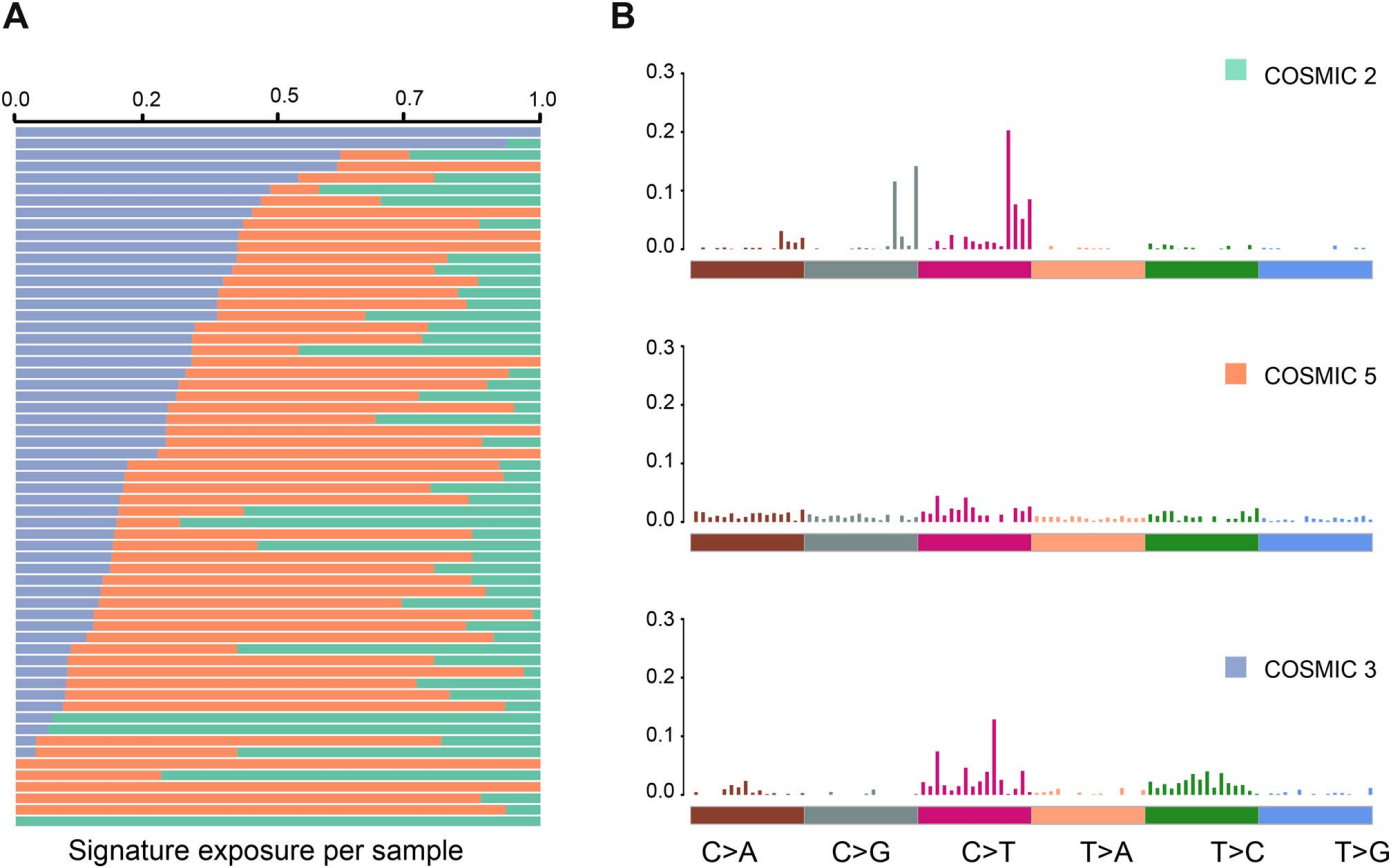
Mutational Signatures in the Swedish BC cohort. (**A**) The relative contribution of each sample to the three mutational signatures found in the Swedish cohort. The COSMIC signature 2 (green) is the APOBEC deaminase signature usually identified in BC. The COSMIC signature 5 (orange) is not clearly associated with a defined process but is found in many cancers. The COSMIC signature 6 (purple) is associated with a defective DNA mismatch repair. (**B**) The substitution motifs that define the three mutational signatures.

To identify the mutational processes responsible for the somatic mutations we observed in the Swedish cohort, we applied an NMF methodology to extract mutational signatures from our SNV set (Fig. 4). The first signature was most identical with the APOBEC cytidine deaminase signature, known as COSMIC signature 2 (C > T, cosine similarity = 0.85); the second was nearly identical to signature 5 (substitutions at ApTpN, cosine similarity = 0.83); and the third was signature 6 (shorter than 3 bp indels at mon/polynucleotide repeats) whose etiology stems from a defective DNA mismatch repair. We also investigated whether the samples with mutations in the top three genes exhibited different signatures. The *PIK3CA*-mutated group mainly had COSMIC signature 12 (of unknown etiology), the *KMT2C*-mutated group COSMIC signature 20 (defective DNA mismatch repair), while the *TP53*-group mainly had the COSMIC signature 15 (defective DNA mismatch repair) and the COSMIC signature 7 (UV exposure).

### Differences in the age groups

Looking at the two age groups (n_young_ = 38; n_old_ = 23) more closely, we observed a higher median mutation rate in the younger group (mut_young_ = 536, median_young_ = 9.5 vs mut_old_ = 234 median _old_ = 8.0 red, Fig. 1A). *PIK3CA, KMT2C* and *TP53* are the top mutated genes in both age groups. In the younger group, *TP53* was mutated in 29% of the patients while only in 9% of the older patients. *PIK3CA* and *KMT2C* mutations had almost equal frequencies in the younger and older groups (*PIK3CA*: 26% vs 30%, *KMT2C*: 21% vs 26%, SF10). The third SMG, *CDH1,* was mutated in both age groups but was more frequently mutated in the older (17%) versus the younger patients (5%, SF10). Notably, *CDH23* mutations were completely absent from the older set while it was mutated in 18% of the patients below 70 years of age. CNAs were seen in both age groups equally (Additional File 1: S.Table 4), but a region on 19p13.11 was deleted more often in the younger group (*p*-value = 0.026), which influenced 169 genes around the targeted gene *CCDC124.*

Based on these observed differences between the two age groups, we investigated if different mutational hotspots were detected more frequently in the younger or older groups. On *PIK3CA*’s longest transcript, both groups carried the well-known hotspot mutation on H1047R and had the same distribution of mutations in the other protein domains. For *KMT2C*, both groups showed mutations in an intra-domain region with no known function, while the older samples had more mutations that lead to a truncated protein just before the PHD domain. *TP53* was mutated mostly in the younger samples while *CDH1* was mutated in the older and lobular cancer samples causing a truncated CDH1 protein (Additional File 7: SF9).

The mutational signatures were relatively similar between the age groups, with APOBEC deaminase signature being the main signature in both. Nevertheless, the contribution to this signature was caused by different genes in the two age groups. In detail, *MAP3K1* and *CDH23* were the genes contributing to the APOBEC signal in the samples under 70 years old, while in the older group above 70 years old, mostly *KMT2C* mutated samples exhibited the APOBEC signature (Additional File 8: SF11).

## Discussion

Sweden has the second highest prevalence of BC in Europe^1^, but the somatic spectrum of BC mutations in Swedish patients has not yet been described. Here, we report the somatic mutational profile of ER + (85%) BC in a homogeneous Swedish cohort. The cohort consisted of mostly ER + (85%) DC tumors while the more aggressive HER2 + or triple negative subtypes were not equally represented by the sample selection. The inclusion of patients that haven’t received neoadjuvant chemotherapy may have contributed to the lack of these molecular subtypes, given that patients in these groups generally undergo neoadjuvant therapy prior to surgical resection of the tumor. Our cohort contained rather older patients than other studies and showed a bimodal distribution of the onset age (median age = 70 year), setting it apart from previous larger studies (median age = 60 year ^9,10,40^). This distribution inspired a form of two distinct age groups (young: under 70 year, old: above 70 year), that were both relatively old (mean _young_ = 57.6, mean _old_ = 83.1) and exhibited similar phenotypic characteristics and amount of mutations (mut_young_ = 536, median _young_ 9.5 vs mut_old_ = 234, median_old_ = 8.0). We speculate that this may be due to the lack of the young onset cases, that usually have a higher tumor grade, involve the more aggressive tumors compared to the older cases^58^ and undergo neoadjuvant therapy that is not commonly given in postmenopausal women^59^. DNA from the tumors and their matched normal fractions was hybridized with a liquid 20.5 Mb array, containing more than 760 genes, and deeply sequenced (mean target coverage 85 × normals, 140 × tumors). The target genes were identified from previous BC studies, both in human and dog. In comparison with other whole exome and whole genome studies^9,10,60^ our capture is definitely smaller, as it is less than an exome, but in some incidences the number of our targeted genes is larger^10^. Also, our array contained genes that were absent from clinical panels^61^ e.g. *KMT2C*, that was mutated in 23% of our cohort*.* When normalized for capture size, our array detected SNVs at roughly the same density (median mut_SWE_ 0.51) as previously published studies (median mut_TCGA:_ 0.68 ^62^), (SF5) and the observed differences can be explained by the array and sample size. All in all, the targeted array was large enough to appropriately fingerprint the cancer genome and provide good quality data to describe the somatic landscape in Sweden.

Despite the small cohort size and the representation of one molecular subtype, our cohort replicates the previously described somatic landscape of breast cancer^9,10,60^, as we report *PIK3CA, TP53* and *CDH1* as SMGs (Fig. 1). Interestingly, although *CDH1* is usually associated with lobular carcinomas^63^, we found it mutated in 10% of the cohort and classified as a SMG but only 50% of the samples with mutations in the genes were in fact lobular carcinomas, with the rest being DC. We report mutations in genes coding for large proteins including *MUC16, MUC17* and *TTN*. While mutations in these genes were reported before^54^ their role in cancer is not clear. Notably, in our cohort a gene reported as less frequently mutated in breast cancer, *KMT2C* (*MLL3*), showed the second highest mutation count. *KMT2C* mutations have been predominantly found in ER + tumors, same as our study^10^. In contrast, *AHNAK9* and *SYNE1* that have been reported to be frequently mutated^10^ were not observed in our top genes; although *AHNAK* was targeted it was mutated in only 4% of the samples while *SYNE1* (downstream of targeted *ESR1*) was not fully captured by the array. We also noted that *KMT2C* mutations were exclusively detected in conjunction with high VAF mutations in the proto-oncogene *PIK3CA*, suggesting the PI3K perturbation might be more important in our BC cohort. PI3K alterations are more common and are followed by all the other processes such as p53 blocking or enhancers activation from lysine methyltransferases. Apart from KMT2C that is a histone modifier protein, ARID1A is also involved in chromatin remodeling as an important part of that SWI/SNF complex (otherwise known as SWI/SNF-Related, Matrix-Associated, Actin-Dependent Regulator Of Chromatin). Together, these two genes are mutated in 27% of the samples, a percentage similar to that of *PIK3CA* (28%), pointing to the importance of chromatin regulators in the somatic landscape of BC. The smaller target size used in this study means that, VAF calculation for the mutations within the top mutated genes is not as accurate as when whole genome sequencing data is used. Nevertheless, the clonality tendencies in this dataset point to *CDH1*, *TP53* and *PIK3CA* as putative drivers but further analysis will be required.

Regarding the CNA findings, targeted sequencing is not the optimal way to identify large events but the genomic breadth of our array was enough to see the tendencies of focal events. *CDK10*, a known tumor suppressor in BC was significantly deleted and *MDM4*, an oncogene in BC, was amplified; both in a large fraction of the samples. Also, the CNA analysis pinpointed an interesting region on chromosome 6 in the Swedish cohort. The genes in that amplified segment are *ABBC10,* a transmembrane transporter with implication on anti-cancer agent transfer and *ZNF318*, coding for the best substrate for deacetylation enzymes (HDAC8). This finding gives room for many hypotheses to be built. For example, while *ZNF318* was not recurrently mutated in our dataset, it was part of the amplifications observed and could suggest a new disease mechanism and drug target where the amplification of ZNF318 affects the deacetylation of ARID1A. It would be interesting to explore whether a non-accessible *ARID1A* through *ZNF318* amplification*,* might be the trigger of an increased *KMT2C* transcription, where errors will lead to accumulation of truncating mutations.

Apart from the expected results that we replicated, there were some discrepancies compared to COSMIC and other studies. The absence of some genes with previously documented role in BC such as *ESR1* may be due to the stringent filtration of variants. *ESR1* was fully targeted but it contained 12 intronic variants which were not included in the SMG estimation. Also, the specific target design explains why some genes were not included e.g. *TBX3 and APC*^9^ part of the transcription regulation pathway and WNT signaling pathway*.* Notably *MY*C and *ERBB2* were among the targeted genes but did not contain many SNVs. However, they were subject to frequent copy number alterations in our cohort (amplified in 65% and 47%, respectively).

To date, the pathways most commonly implicated in ER + breast cancers are AKT signaling (including *PIK3CA*), DNA damage and apoptosis (including *TP53*) and transcription regulation (containing *GATA3, TBX3*). Based on the percentage of mutations in the genes involved, we report that chromatin function (*KMT2C 23%*, *ARID1A 5%*) are more affected than the transcription regulation (*PIK3CA* 28%, *KMT2C* 23%, *TP53* 21%, *MAP3K1 10*%, *GATA3* 4%) and might be more important in the subset of tumors we analyzed. We also contrasted our observations to the enrichment of mutations within the TCGA oncogenic signaling pathways. There, we observed that RTK-RAS (17 out of 87 genes, in 25 samples) and the TP53 pathway (2 out of 6 genes, in 14 samples) appeared to be the most commonly affected in our cohort, followed by PI3K and TGF-beta pathways (data not shown).

We also noted that the mutated positions in the top genes have been observed before with a mutated catalytic domain in *PIK3CA* and a lot of known missense mutations with carcinogenic properties in *TP53* and the novel mutations were not recurrent, just found in one or two samples. For *KMT2C*, we noted a large fraction of truncating mutations many of which were between the 5′ and 3′ protein domains and could be expected to lead to a shorter protein which would be lacking the PHD domain that is found to be the one regulating which enhancers will be monomethylated (Fig. 3C). As a key regulator of ERα activity, disrupted KMT2C contributes to hormone driven breast cancer proliferation^64^ that might be more obvious in ER + BC.

Investigation of the underlying mutational processes in these tumors identified three different mutational signatures in our cohort. COSMIC signature 2 is attributed to AID/APOBEC deaminase activity and is linked with breast cancer germline mutations, while COSMIC signature 6 is associated with a defective DNA mismatch repair mechanism. This could possibly explain why we see mutations in genes of DNA repair pathway and chromatin regulation such as *KMT2C* and *AR1DIA*. The COSMIC signature 5 does not have a known etiology but is shows strand bias for T > C substitutions as is seen in multiple cancer types.

The bimodal age distribution of the Swedish breast cancer cohort allowed us to look at both middle-aged and old breast cancer patients. In previous reports, all ER + cancers appear with more SNVs, but in this study we saw that specifically the women below 70 accumulate more SNVs. Also, mutations in cadherins *CDH1* and *CDH23* were different among the two groups. *CDH1* mutations were more common in the old group giving them the characteristic of late onset, alongside with the known *CDH1* link with the lobular carcinoma phenotype. *CDH23* mutations were completely missing from the older set possibly revealing a new role for this cadherin gene in ER + BC. Notably, *CDH23* was the gene from the canine mammary GWA study that had the largest number of intronic variants. In addition, we did not notice any correlation between the variation observed in the normal fraction of the tumors (germline) and the set of true somatic mutations, most probably due to the rather old patients examined here. Further follow up of our results, incorporating the non-coding mutations as well will be valuable for a more complete characterization of this Swedish breast cancer cohort.

## Conclusions

Our study, the first BC tumor/normal sequencing study in Sweden, consisted of a younger and an older subgroup. The results, despite the small size, replicated a similar mutational rate and key genes found for BC genes in previous studies. Our study also revealed that the histone modification gene *KMT2C* was frequently mutated in many samples especially in the older group. Many *KMT2C* mutated samples had a truncated PHD-domain, suggesting that more functional work should be performed to fully understand the consequences of a non-functional KMT2C protein. In addition, age affected the mutational landscape of the ER + tumors with possible clinical implications. Taken together, this study describes the fingerprint of the somatic breast cancer landscape in Sweden and its findings point towards several potential mechanisms underlying the observed differences.

## Supplementary information

Supplementary file1

Supplementary file2

## Data Availability

Annotated, unfiltered, mutation and CNV data, along with R code related to this study, are available upon reasonable request.
