# Author Correction: Targeted sequencing reveals the somatic mutation landscape in a Swedish breast cancer cohort

**DOI:** 10.1038/s41598-021-84929-9

**Published:** 2021-03-01

**Authors:** Argyri Mathioudaki, Viktor Ljungström, Malin Melin, Maja Louise Arendt, Jessika Nordin, Åsa Karlsson, Eva Murén, Pushpa Saksena, Jennifer R. S. Meadows, Voichita D. Marinescu, Tobias Sjöblom, Kerstin Lindblad-Toh

**Affiliations:** 1 Science for Life Laboratory, Department of Medical Sciences, Uppsala University, Uppsala, Sweden; 2 Science for Life Laboratory, Department of Immunology, Genetics and Pathology, Rudbeck Laboratory, Uppsala, Sweden; 3 Science for Life Laboratory, Department of Immunology, Genetics and Pathology, Rudbeck Laboratory, Uppsala, Sweden; 4 Department of Veterinary Clinical Sciences, Faculty of Health and Medical Sciences, University of Copenhagen, Copenhagen, Denmark; 5 Science for Life Laboratory, Department of Medical Biochemistry and Microbiology, Uppsala University, Uppsala, Sweden; 6 Department of Clinical Pathology and Genetics, Sahlgrenska University Hospital, Gothenburg, Sweden; 7 Broad Institute of MIT and Harvard, Cambridge, MA USA

Correction to: *Scientific Reports* 10.1038/s41598-020-74580-1, published online 09 November 2020

This Article contains an error in the Discussion section where,

“For *KMT2C*, we noted a large fraction of truncating mutations many of which were between the 5′ and 3′ protein domains and could be expected to lead to a shorter protein which would be lacking the PHD domain that is found to be the one regulating which enhancers will be monomethylated (Fig. 3C).”

should read:

“For *KMT2C*, we noted a large fraction of truncating mutations which could be expected to lead to a shorter protein lacking the PHD domain, and in turn, the deregulation of enhancer methylation (Fig. 3C).”

