# Supplementary material for: Targeted sequencing reveals the somatic mutation landscape in a Swedish breast cancer cohort
Source: Sci Rep. 2020 Nov 9;10:19304. doi: 10.1038/s41598-020-74580-1 (PMC7653953; doi:10.1038/s41598-020-74580-1)
Supplement: Supplementary file 1 — Supplementary file1 [file 41598_2020_74580_MOESM1_ESM.pdf]

Targeted sequencing reveals the somatic mutation landscape in a Swedish breast cancer cohort.

Argyri Mathioudaki[1], Viktor Ljungström[2], Malin Melin[3], Maja Arendt[1,4], Jessika Nordin[1], Åsa Karlsson[1], Eva Murén[1], Pushpa Saksena[5], Jennifer R. S. Meadows[1], Voichita D. Marinescu[1], Tobias Sjöblom[2], Kerstin Lindblad-Toh[1,6]

[1] Science for Life Laboratory, Dept. of Medical Biochemistry and Microbiology, Uppsala University, Uppsala, Sweden
[2] Science for Life Laboratory, Dept. of Immunology, Genetics and Pathology, Rudbeck Laboratory, Uppsala, Sweden
[3] Science for Life Laboratory, Dept. of Immunology, Clinical Genomics, Rudbeck Laboratory, Uppsala, Sweden
[4] Department of Veterinary Clinical Sciences, Faculty of health and medical sciences, University of Copenhagen, Copenhagen, Denmark
[5] Department of Clinical Pathology and Genetics, Sahlgrenska University Hospital, Gothenburg, Sweden
[6] Broad Institute of MIT and Harvard, Cambridge, Massachusetts, United States of America

Supplementary Figures

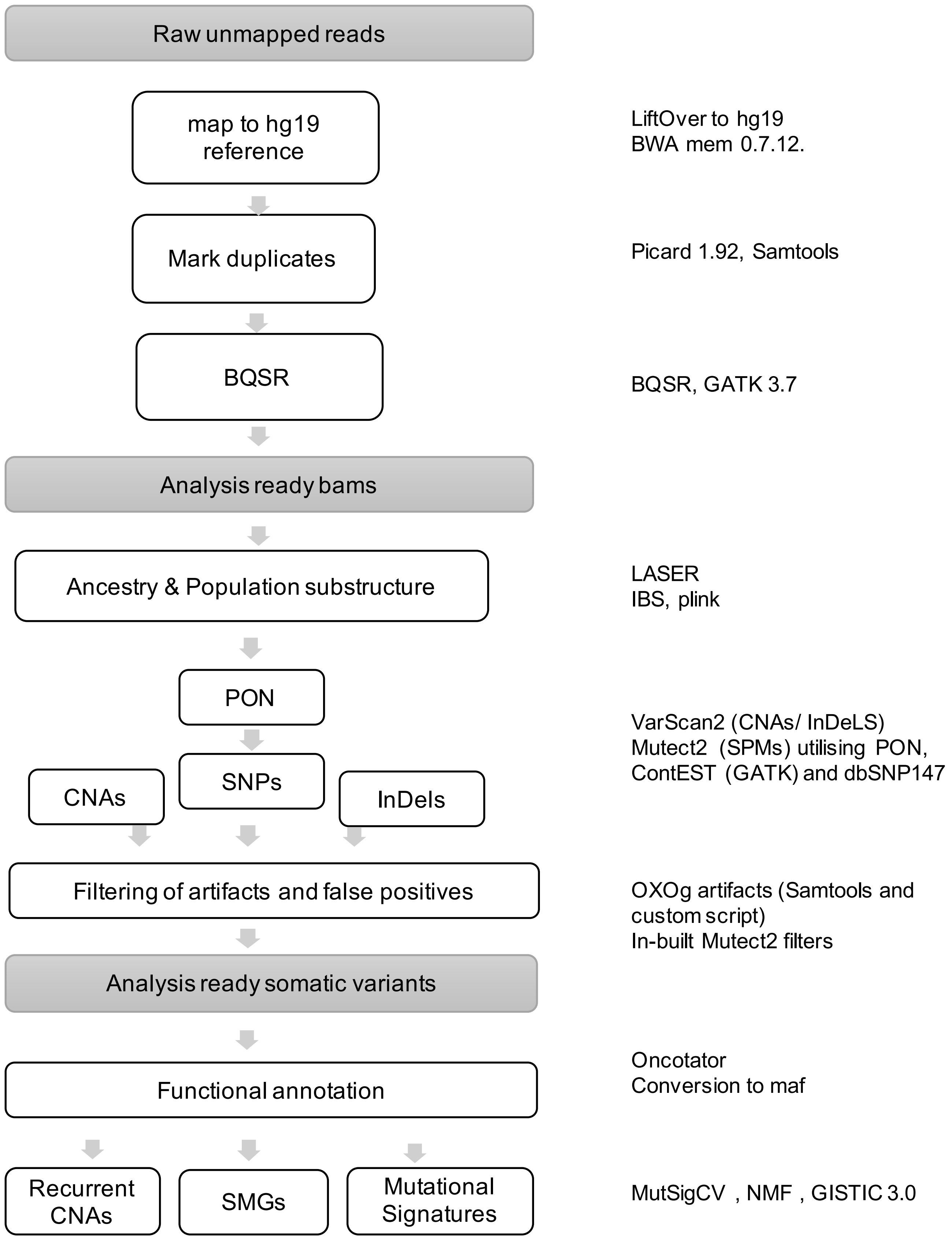

Supplementary Figure 1. The different pipeline steps and tools utilized in the generation of the somatic dataset.

Overview of the main pipeline steps and tools of data analysis from raw sequences to recurrent somatic events and mutational signatures. All the samples underwent a common pre-processing step and ancestry analysis. Then, CNA and InDels calling utilized VarScan2 while SNV detection was performed with MuTect2 and required simultaneous analysis of the tumor with its matched normal pair. Filtration and functional annotation were performed on SNVs followed by identification of recurrent somatic events on both CNAs and SNVs.

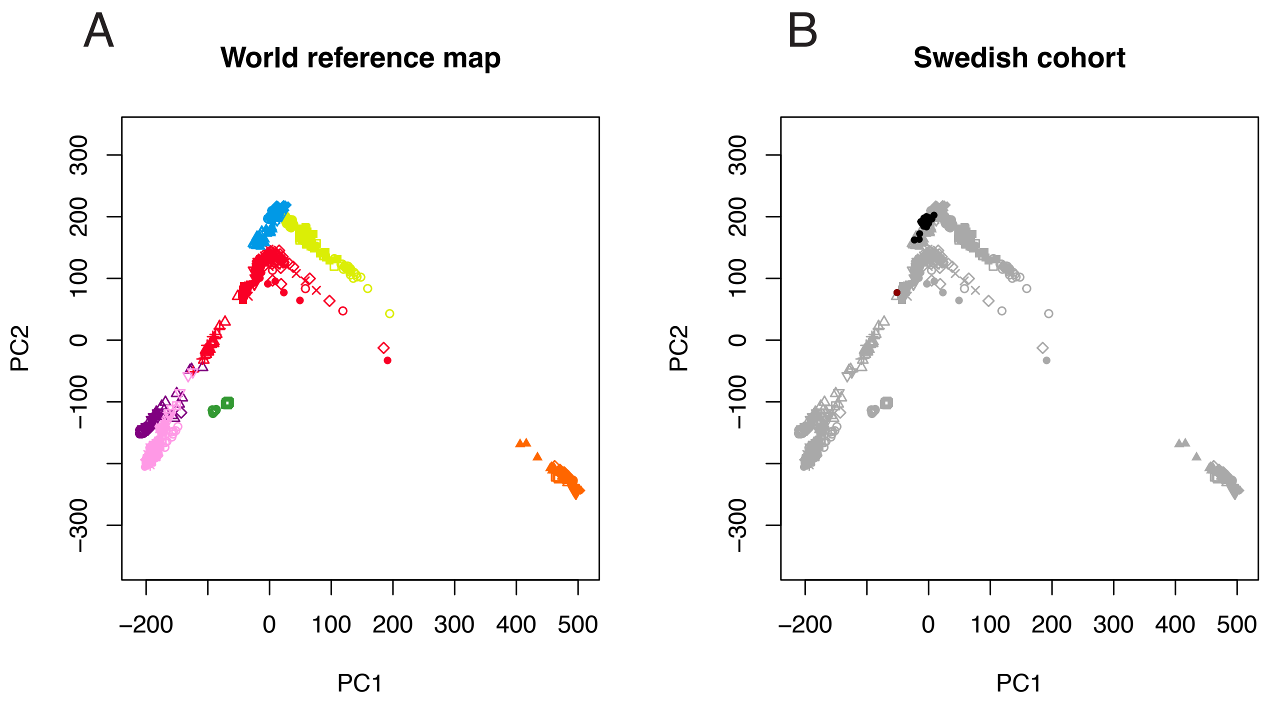

Supplementary Figure 2. LASER analysis of the European ancestry of the samples in the Swedish BC cohort.

As part of quality control, ancestry assessment was performed using LASER (5) on the normal fraction of the tumor-normal pairs to determine putative outliers within our sequenced samples. The plots show the two first principal components which were used to estimate Swedish ancestry based on the markers in the Human Genome Diversity Project (HGDP) reference panel. A The world reference map according to HGDP. Markers that appear with light blue and predict European ancestry are shown based on PC1 and PC2. B Projection of the Swedish cohort upon the reference map (shown in grey) to estimate ancestry. Swedish samples were removed if they were not within the European coordinates (± 3 SDs) and are marked with red panel while the samples retained after the ancestry control are colored with black in this panel.

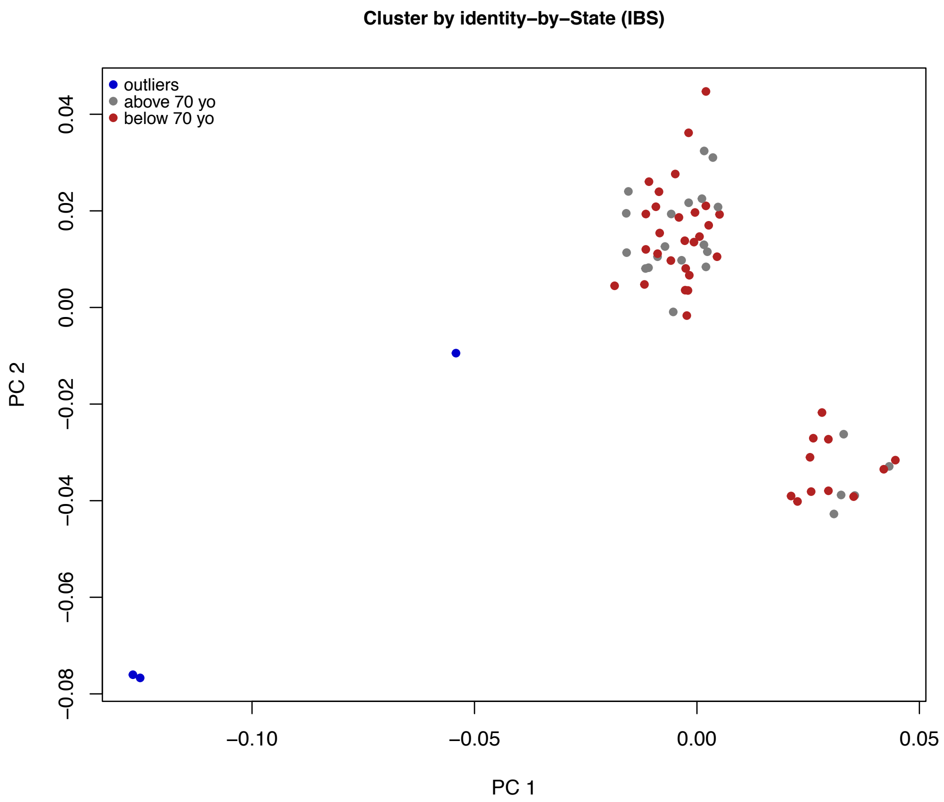

Supplementary Figure 3. IBS clustering of normal samples.

We checked for population stratification utilizing data from the all 65 sequenced normal samples. After removal of the regions that are in high LD in the European population, we plotted the clustering that was based on identity by descent estimation. Putative outliers (blue) that were not in the main sample clusters were removed from further analysis. The remaining two clusters could not be explained by ancestry, phenotypic criteria, amount of data or sequencing metrics. In the graph it is illustrated, that age is not a factor for clustering (young samples shown with grey, old samples shown with red). We analyzed whether some specific genomic regions were influencing these principal components for this clustering and we saw that there is no variant load in specific markers that could influence the observed clustering.

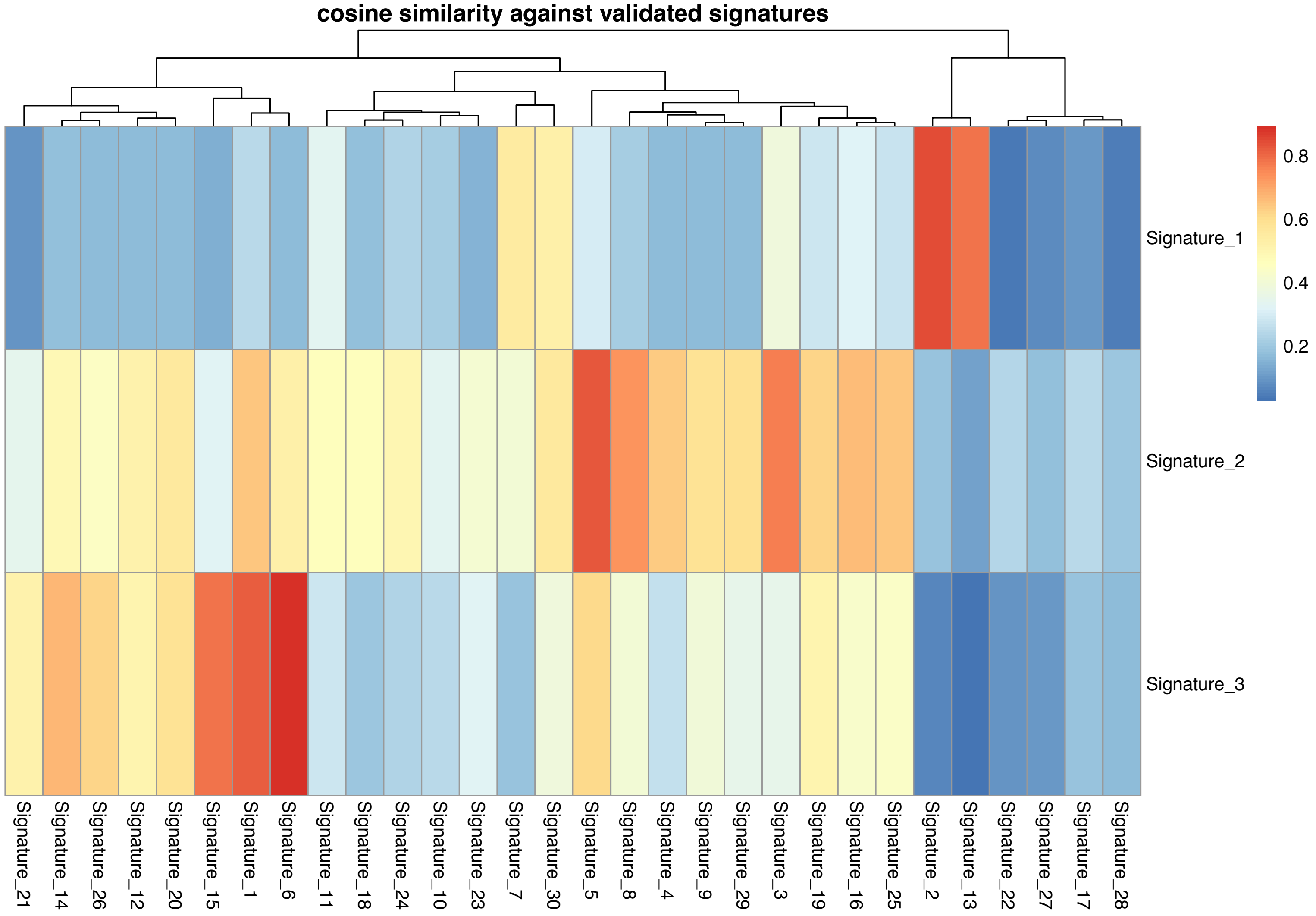

Supplementary Figure 4. Cosine similarity of extracted mutational signatures with validated COSMIC signatures.

Using NMF we compared the signatures extracted from the BC mutational set against the experimentally validated 30 signatures (See http://cancer.sanger.ac.uk/cosmic/signatures for details.) The three horizontal bars represent the top three signatures identified in our cohort and the vertical bars are the 30 known mutational signatures in COSMIC. The different coloring schemes reveals how identical they are, with red showing high concordance. The identified Signature_1 was most similar (red) to COSMIC Signature_2 (cosine-similarity: 0.85) whose etiology is due to APOBEC Cytidine Deaminase (C>T). Signature_2 was most similar to the COSMIC Signature_5 (cosine-similarity: 0.83) whose etiology is unknown but is widespread in all cancers. Finally, the Signature_3 was most similar to the COSMIC Signature_6 (cosine-similarity: 0.893) whose etiology is due to defective DNA mismatch repair.

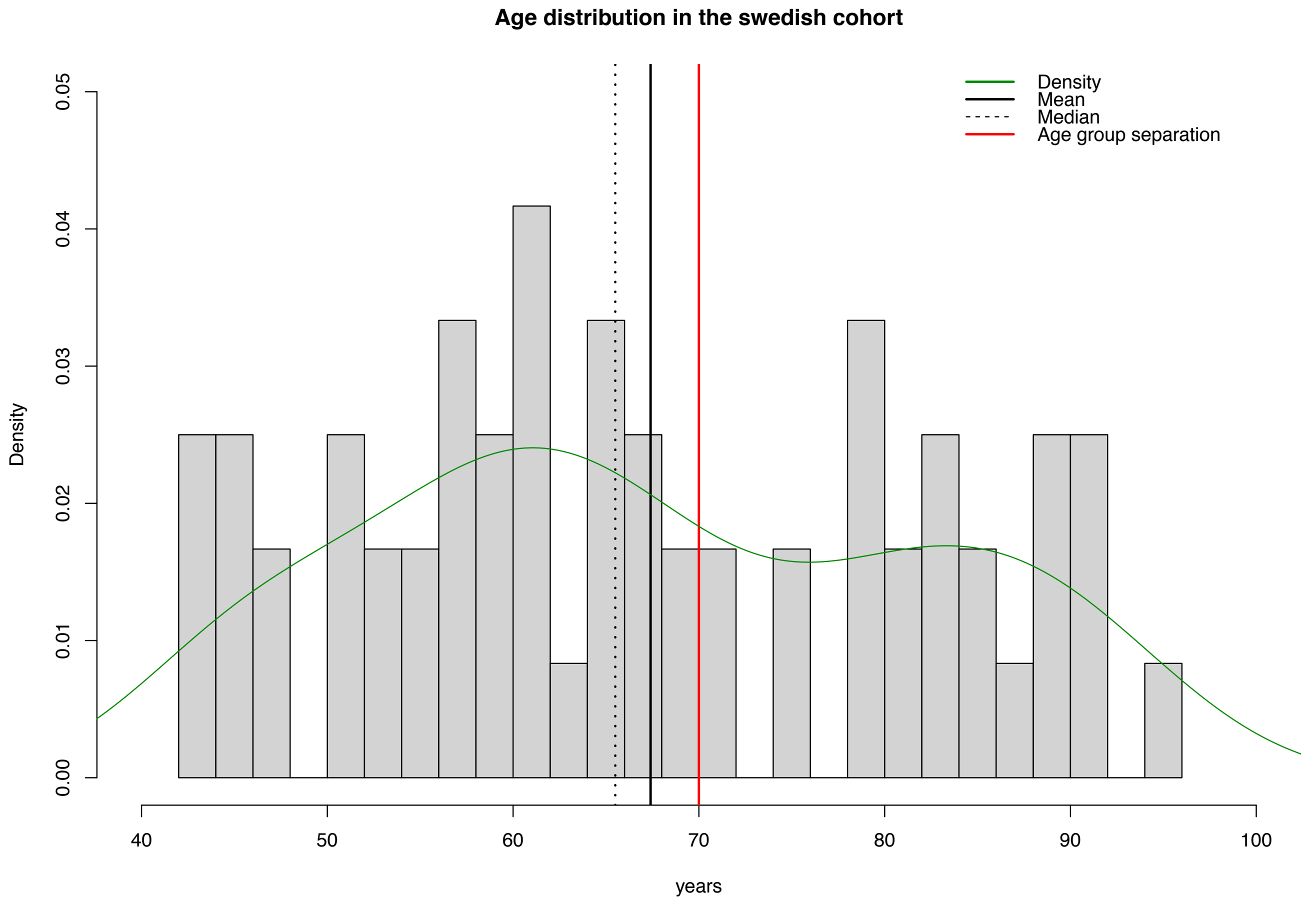

Supplementary Figure 5. Histogram of the age of onset distribution in the Swedish cohort.

Illustration of the onset age in the 61 samples after ancestry and quality control. According to the density line, the distribution appears as bimodal with a mean of 67.4 (black line) and a median of 65.5 years (dashed line). Age clustering with a gaussian finite mixture model fitted by EM algorithm proved that indeed two age clusters exist in our dataset. That observation led to a further division of the cohort into two separate age groups i) below 70 years old (n = 38) and ii) above 70 years old (n = 23) as shown in the illustration with the red line.

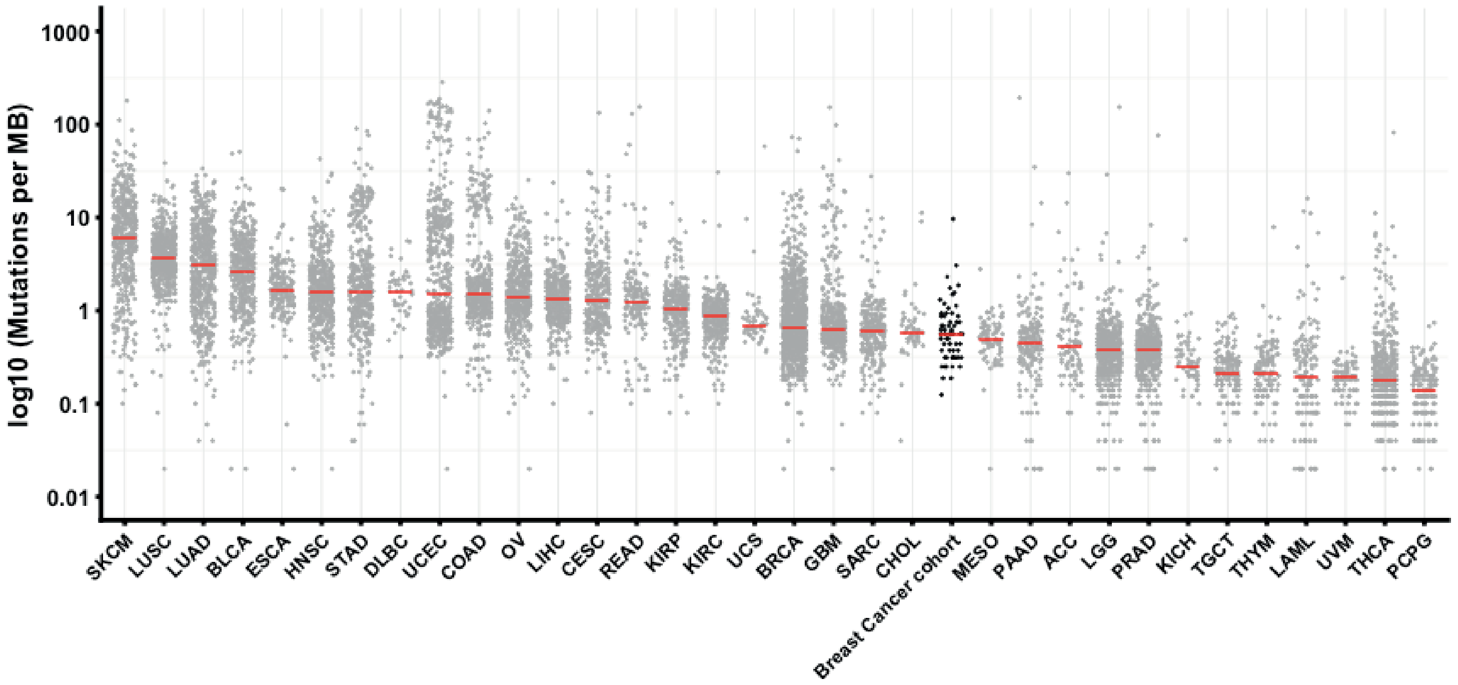

Supplementary Figure 6. Swedish BC targeted cohort compared to TCGA BC cohort

The graph represents the distribution of variants compiled across 33 TCGA landmark cohorts and the Swedish BC cohort, normalized for the size of coding target. The different median mutations per project are marked with red. The distribution in the Swedish cohort (n = 61) is different from the TCGA breast cancer cohort (n = 1,044), but the observed differences are mainly due to capture and sample size.

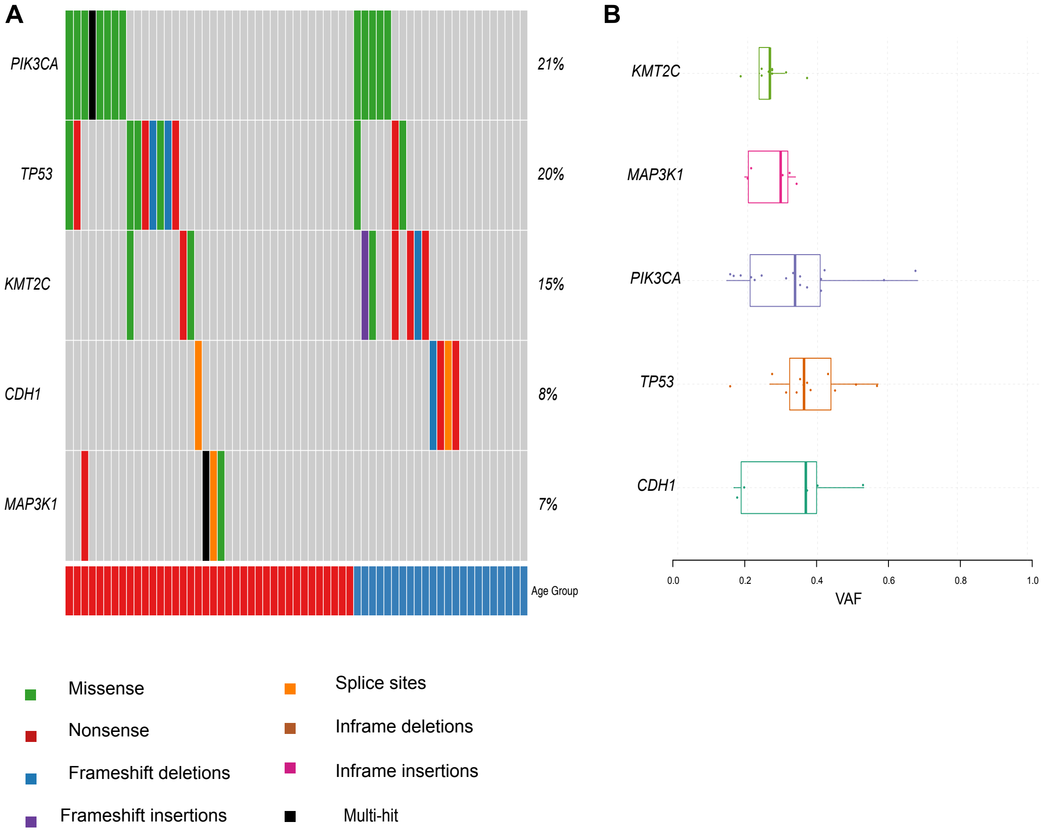

Supplementary Figure 7. VAF filtered onco-plot of somatic SNVs and CNAs in the Swedish breast cancer cohort.

A Brick plot of mutations in the VAF filtered (> 15%) somatic SNV and indels set and their distribution across genes. The samples are ordered according to which age group they belong to below (blue) or above 70 years old (grey) while receptor information also shown. Utilizing MutSigCV algorithm, it we found that the significantly mutated genes are *PIK3CA, TP53, CDH1* (q-value cutoff 0.01, shown with *), which were estimated to be mutated in these tumors more than the calculated background mutation rate. B VAF boxplot for the genes with the clusters with the highest VAF, which are in decreasing order: *CDH1, TP53, PIK3CA, MAP3K7, KMT2C.*

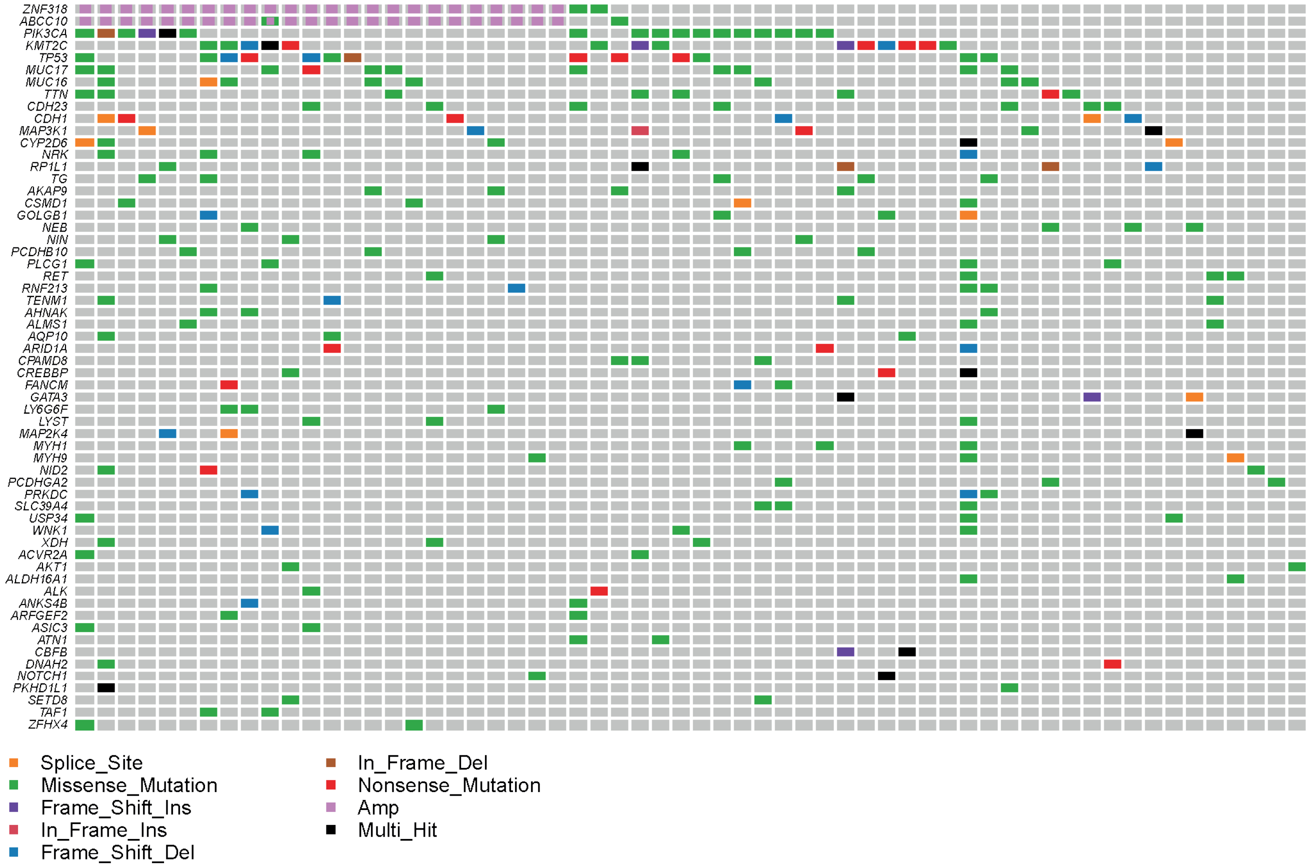

SupplementaryFigure 8. Somatic coding SNVs/indels and CNAs in the Swedish cohort.

The genes that contain the majority of the point mutations and indels are not subject to copy number alterations while two genes that show many amplifications, *ABCC10* and *ZNF318* have also a few missense mutations. The rest of the CNAs was not visualized here as the genes that they affected do not contain coding mutations.

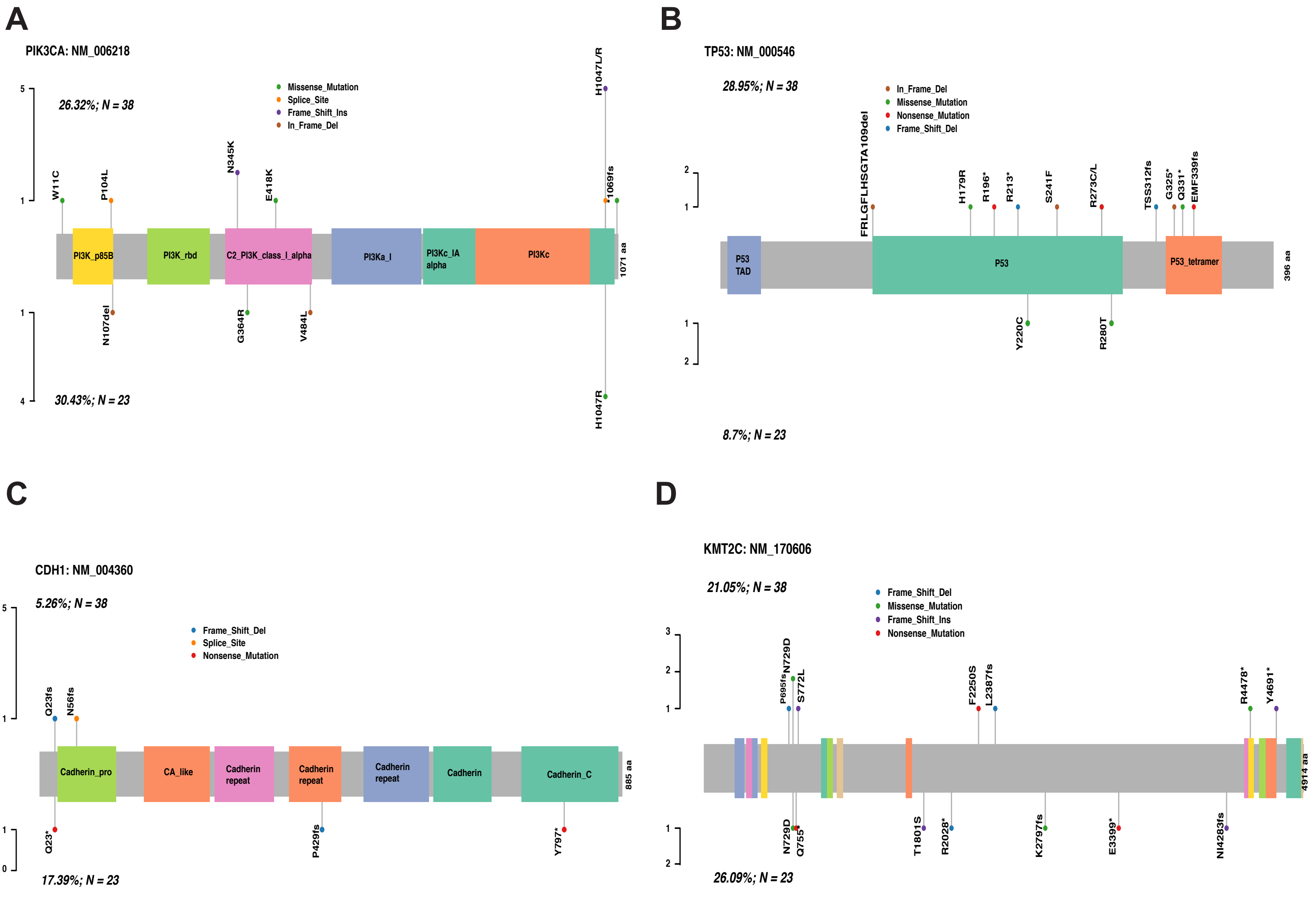

Supplementary Figure 9. Mutations on the most recurrently mutated genes as seen in the different age groups.

All the lollipop plots above, illustrate on the top panel the mutations from the young group (under 70 years old) and on the lower panel the mutations from the older group (over 70 years old). A Lollipop plot of *PIK3CA,* with somatic mutation rate 26.3% in the younger and 30.5% in the older patients. The H1047R hotspots was seen in both age groups. H1047R is a well-known BC hotspot, also identified in the Swedish cohort. B *TP53* showed a mutation rate 29% in the younger patients and only 8% in the older patients. No hotspots were identified in the cohort but a big proportion of mutation on the p53 domain mainly in the younger group. C *CDH1* had a mutation rate 17% in the older age group and only 5% in the younger group, where the CA like domain was affected by one mutation. D *KMT2C* showed a somatic mutation rate 21% in the younger patients and 26% in the older cases. PHD domain was not affected by the mutations but mutations upstream might affect its correct formation. A hotspot with two mutations was reported in the younger patients while mutations in an unknown function domain were reported in the older group.

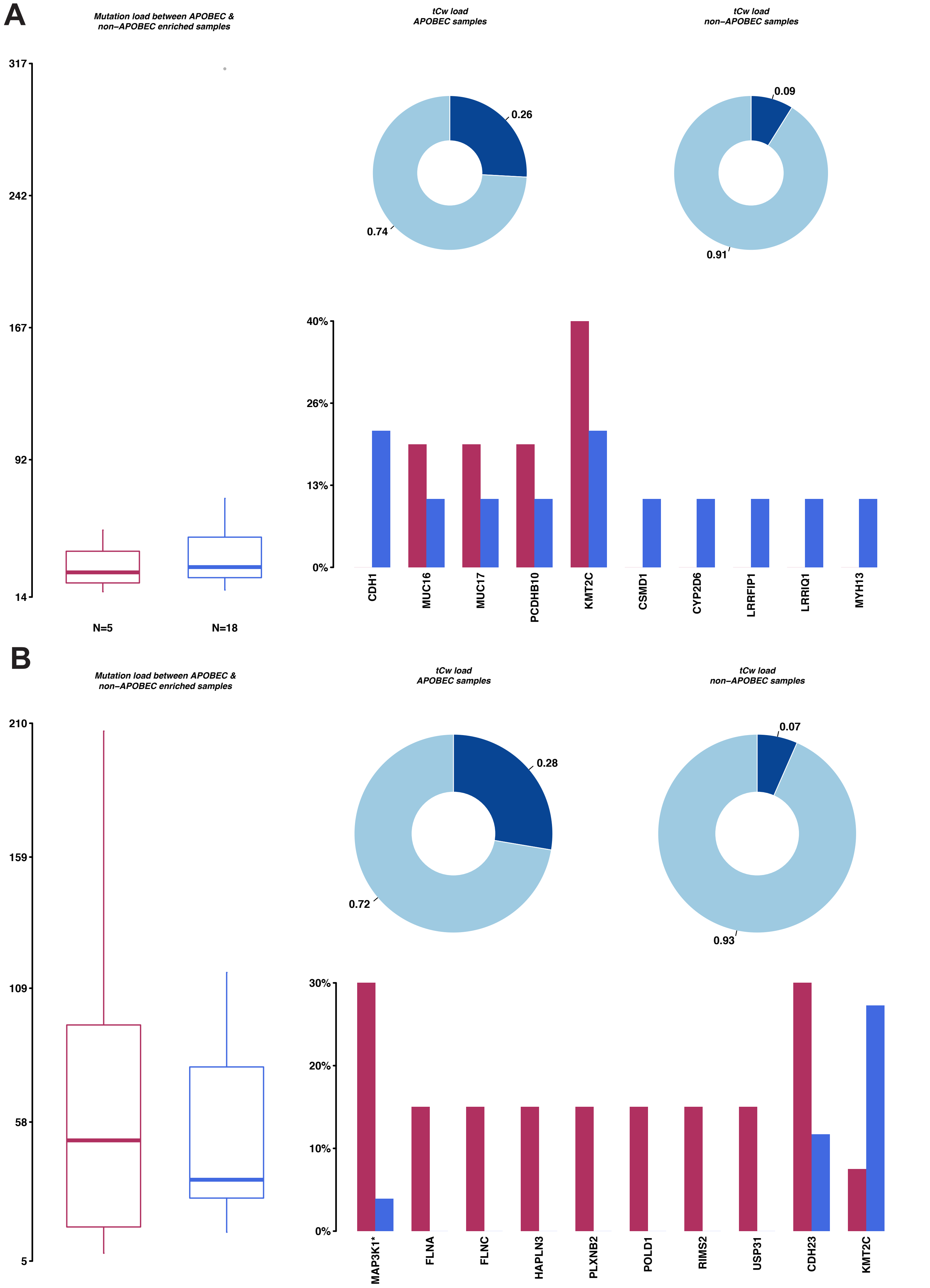

Supplementary Figure 11. APOBEC differences in the two age groups.
It is illustrated which genes were driving the APOBEC signature (pink bars), and which did not (blue bars) and weather these were different among the two age groups A older samples (above 70 years old) and B younger samples (below 70 years old).
